# First Dose of BNT162b2 mRNA Vaccine in a Healthcare Worker Cohort Is Associated With Reduced Symptomatic and Asymptomatic Severe Acute Respiratory Syndrome Coronavirus 2 (SARS-CoV-2) Infection

**DOI:** 10.1093/cid/ciab351

**Published:** 2021-04-23

**Authors:** Patrick J Lillie, Paul O’Brien, Michelle Lawtie, Steve Jessop, Nicholas J W Easom, Russell Patmore

**Affiliations:** 1 Infection Research Group, Department of Infection, Hull Teaching Hospitals, Hull, United Kingdom; 2 Department of Pharmacy, Hull Teaching Hospitals, Hull, United Kingdom; 3 Staff Testing Service, Hull Teaching Hospitals, Hull, United Kingdom; 4 Lead nurse vaccine rollout team, Hull Teaching Hospitals, Hull, United Kingdom; 6 Department of Hematology, Hull Teaching Hospitals, Hull, United Kingdom

**Keywords:** SARS-CoV-2, COVID-19: vaccination, BNT162b2, lateral flow test

## Abstract

Over the first 2 months of 2021, vaccination coverage of staff at Hull Teaching Hospitals with BNT162b2 increased from 8.3% to 82.5% and was associated with a significant reduction in symptomatic and asymptomatic severe acute respiratory syndrome coronavirus 2 (SARS-CoV-2) cases. The proportion of positive lateral flow tests from asymptomatic screening was maintained over this period.

The rollout of vaccination against severe acute respiratory syndrome coronavirus 2 (SARS-CoV-2) in the United Kingdom has included healthcare workers as a priority group. The efficacy of currently licensed vaccines against symptomatic disease is well documented from clinical trials [1, 2]; however, there are fewer data with regard to asymptomatic shedding of virus, or the changing pattern of symptoms of those infected as vaccination programs progress. Hull University Teaching Hospitals has used the Pfizer-BioNtech vaccine (BNT 162b2) for staff vaccination and has been recording testing for SARS-CoV-2 by both nucleic acid amplification tests (NAAT) for all symptomatic staff and specific screening, together with routine (asymptomatic) lateral flow device (LFD) testing of all clinical staff. Here we present the effect of the vaccine rollout on staff polymerase chain reaction (PCR) and LFD positivity across symptomatic and asymptomatic groups.

## METHODS

**Figure 1.**
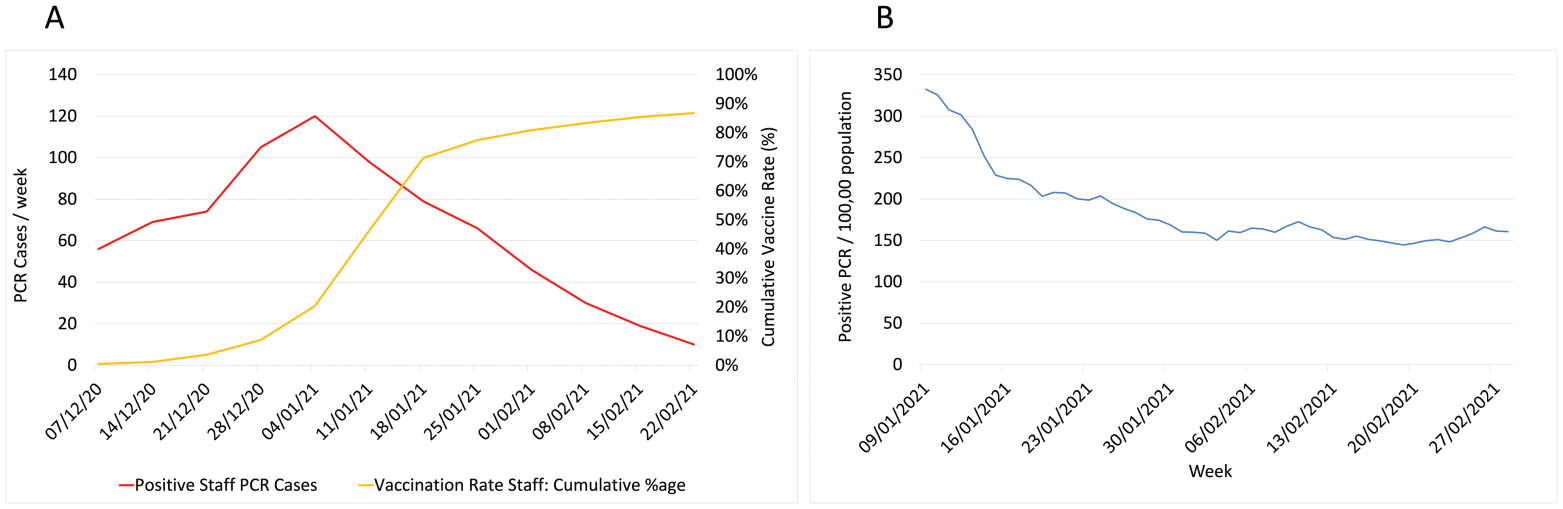
*A*, Vaccination coverage and PCR positive test numbers. *B*, Positive PCR tests / 1 000 000 in Kingston upon Hull during study period. Abbreviation: PCR, polymerase chain reaction.

Vaccination with BNT 162b2 began in staff on 9 December 2020, with a planned acceleration in staff vaccination from 28 December 2020 (Figure 1). Vaccination was carried out as per guidelines from Public Health England (PHE) and the Department of Health [3]. As per guidance from the Joint Committee on Vaccination and Immunization (JCVI) [3], initial doses have been administered with the booster dose planned for 10–12 weeks post first vaccination.

Since April 2020, symptomatic staff testing for possible coronavirus disease 2019 (COVID-19) disease has been performed. In addition to testing being triggered by classical symptoms of COVID-19 as per PHE guidance [4] (fever, cough, shortness of breath, loss of smell/taste), we have tested healthcare workers (HCWs) with milder atypical symptoms (nasal symptoms, sore throat, diarrhea, headache, myalgia/arthralgia) as well as targeted screening during outbreaks and routine screening of staff working on the stem cell transplant unit (116 staff members). Staff contact the hospital testing team and attend within 24 hours for a combined nose and throat swab, which is processed internally by the virology department. A variety of molecular assays are used, with the majority of routine tests being performed using the Panther Fusion (Hologic) assay. Clinical staff in all areas have been performing and electronically self reporting their own LFD tests (Innova) as twice weekly asymptomatic testing since 27 November 2020. Data on symptomatic/asymptomatic/LFD testing are presented from the week beginning 4 Jan 2021. We are unable to directly link vaccination status to NAAT or LFD testing among staff. All data were collected during routine working practice, and no ethical approval was deemed necessary, with the COVID-19 steering group for the trust approving data collection and usage. Statistical testing was performed using GraphPad Prism software version 6.

## RESULTS

**Figure 2.**
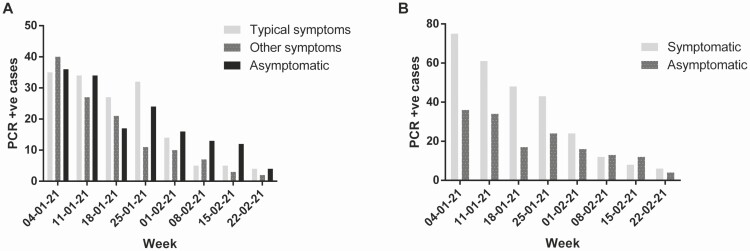
*A*, PCR positive cases by symptom type. *B*, All symptomatic vs asymptomatic PCR cases. Abbreviation: PCR, polymerase chain reaction.

By 4 January 2021 827 (8.3%), staff had received their first dose of BNT 162 b2, increasing to 8243 (82.5%) by the end of the week of 22 February. NAAT-proven cases of SARS-CoV-2 among staff reduced from 120 cases in the week of 4 January to 10 cases in the week of 22 February (Figure 1). There was a significant negative correlation between cumulative vaccination and PCR positive cases (Pearson’s R = −0.9061, *P* = .0019), along with a marked correlation between symptomatic PCR testing rates and vaccine coverage (Pearson’s R = −0.8972, *P* = .0025). The number of staff self-isolating as positive for SARS-CoV-2 on 11 January was 325, dropping to 91 on 23^r^ February (72% decrease), with a 68% reduction in staff isolating due to a positive household contact (100 decreasing to 32), with a smaller decrease in the same time period in non-COVID-19 absence from work (413 decreasing to 328, a 20.58% decrease). Figure 2A and 2B show the relationship between symptomatic / asymptomatic PCR positive cases and date as a marker of vaccine coverage, with a significant correlation between cumulative vaccination and reduction in all categories, *P* < .005 for symptomatic and asymptomatic. Lateral flow testing rates declined over the period from 4269 tests the week of 4 January to 1755 tests recorded in the week of 22 February; however, the proportion of LFD tests reading positive was maintained over this time (R = .7012, *P* = .0527) with between 1.3% and 0.45% being positive. Among vaccinated staff, 13 positive LFD tests were confirmed by NAAT testing, all within 14 days of their vaccination. Figure 1B shows the rate of SARS-CoV-2 positive testing in Kingston upon Hull (data obtained from PHE website) during the same time period, showing an initial rate of 332.6 / 100 000 on 9^t^ January 2021 to 144.4 /100 000 on 19 February 2021 and plateauing from that point. Very few cases of lineage B1.1.7 were present during this period on available whole genome sequencing data, and no other variants of concern were reported.

## DISCUSSION

HCWs are at high risk of being exposed to SARS-CoV-2 [5], and vaccination of this group is a priority. Together with recent data from the SIREN study across UK healthcare [6], these data highlight the high level of efficacy of the BNT162b2 vaccine against both symptomatic and asymptomatic infection. We have found that this effect is noted in atypical as well as typical symptoms of COVID-19, hopefully allowing current testing and screening programs to maintain criteria.

Asymptomatic testing with LFDs has decreased over time, but the proportion of positive tests has remained relatively static, suggesting that sensitivity may be maintained post-vaccination. As the testing by LFD is for routine asymptomatic screening, it is unlikely that the reduction in test reporting is a direct consequence of the vaccination program but may reflect both “testing fatigue” or perceived protection from the vaccination. Under-reporting, particularly of negative tests, is a possible source of bias in these data. In healthcare settings where positive LFD tests can be confirmed by NAAT testing, these tests may retain utility in a vaccinated group.

Limitations of our data include only short-term effectiveness being ascertained; it is an ecological study as opposed to any controlled data; and it is data from a single center in the context of a national vaccine rollout program. Therefore, applicability to other settings is likely to be limited.

In conclusion, single-dose BNT162b2 has been associated with a significant decrease in positive PCR for SARS-CoV-2 in both symptomatic and asymptomatic HCWs, with the most dramatic effect on symptomatic illness. Utility of LFD testing positive test rate seems to be unaffected by the vaccine rollout and may continue to provide a useful screening tool in vaccinated cohorts.

## Note

***Potential conflicts of interest.*** J. W. E. has received lateral flow test kits from Mologic Ltd for an unrelated project. All other authors report no potential conflicts. All authors have submitted the ICMJE Form for Disclosure of Potential Conflicts of Interest. Conflicts that the editors consider relevant to the content of the manuscript have been disclosed.
